# The experiences of psychiatric patients, their caregivers and companions in upholding patient dignity during hospitalization: A qualitative study

**DOI:** 10.1111/hex.13799

**Published:** 2023-06-18

**Authors:** Amirahmad Shojaei, Yosra Raziani, Colleen Bernstein, Ali Asgari, Farshid Alazmani‐Noodeh, Mohammadreza Arab, Hadi Ranjbar

**Affiliations:** ^1^ Medical Ethics and History of Medicine Research Center, Department of Medical Ethics, School of Medicine Tehran University of Medical Sciences Tehran Iran; ^2^ Department of Nursing Al‐Mustaqbal University College Hillah Babylon Iraq; ^3^ Department of Psychology University of the Witwatersrand Johannesburg South Africa; ^4^ Infectious Diseases Research Center AJA University of Medical Sciences Tehran Iran; ^5^ Critical Care Nursing Department, Faculty of Nursing AJA University of Medical Sciences Tehran Iran; ^6^ Department of Surgery, School of Medicine Bam University of Medical Sciences Bam Iran; ^7^ Mental Health Research Center, Psychosocial Health Research Institute Iran University of Medical Sciences Tehran Iran

**Keywords:** dignity, hospitalization, negative guardianship, psychiatric patient

## Abstract

**Introduction:**

The quality of care and patient satisfaction is closely linked with dignity, which is a crucial component of therapy and care. However, there is very little study on dignity in the context of mental health care. Planning for ongoing patient care might benefit from an understanding of the notion of dignity by exploring the experiences of patients, caregivers and companions of patients who have a history of hospitalization in mental health institutions. To retain patients' dignity while they were being treated in mental wards, this study sought to understand the experiences of patients, caregivers and companions of patients.

**Materials and Methods:**

This investigation was qualitative. Semistructured interviews and focus groups were utilized to collect the data. The purposeful sampling method was employed for participant recruitment, which continued until data saturation. Two focus group discussions and 27 interviews were conducted. Participants included 8 patients, 2 patients' family members (companions), 3 psychologists, 4 nurses and 11 psychiatrists. Two focus group discussions were held with seven family members or companions of patients. Thematic analysis was used for data analysis.

**Results:**

The primary theme that emerged was the infringement of patients' dignity, through negative guardianship, dehumanization and violations of their rights. Subthemes included dehumanization, worthlessness and namelessness, patient rights violations and stripping patients of authority.

**Conclusion:**

Our results suggest that, regardless of the severity of the illness, the nature of psychiatric illness significantly compromises patients' dignity. Mental health practitioners, due to their sense of guardianship, may unintentionally treat patients with mental health disorders, thus compromising the patient's dignity.

**Patient or Public Contribution:**

The research team's experiences as a psychiatrist, doctor and nurse informed the study's objectives. Nurses and psychiatrists who work in the healthcare industry designed and conducted the study. The primary authors, who are healthcare providers, collected and analysed the required data. Furthermore, the entire study team contributed to the writing of the manuscript. Study participants were involved in the data collection and analysis.

## 1 INTRODUCTION

Mental illnesses are a crucial global health concern,^1^ affecting at least one in three individuals during their lifetime.^2^ A meta‐analysis revealed that 27% of adults in European countries experienced mental disorders within the past year,^3^ while some studies in Iran reported a prevalence of over 29%.^4^ Preserving patients' human dignity while providing quality care is a fundamental responsibility of any country's healthcare system,^5^ particularly because individuals with mental disorders are vulnerable to losing their human dignity.^6^ Dignity and respect are two main parts of the patient–caregiver relationship.^7^ To underscore the significance of preserving dignity in mental health care, the World Mental Health Federation designated 2015 as the Year of Mental Health Dignity.^8^

The term ‘dignity’ is defined as the treatment of a person with honour, integrity and courtesy in a manner that is not condescending or patronizing, and with equality and the same respect as any other individual desires. It is a mutual respect that cannot be achieved without the participation of both parties. Patient dignity entails recognizing each person's uniqueness and emotions and granting them control over their decisions and actions.^9^, ^10^ Additionally, dignity encompasses how one presents oneself to others in terms of physical appearance and personal conduct. Ultimately, dignity is reflected in the attitudes and behaviour of others toward an individual. The framework of human dignity posits that dignity is a subjective notion that pertains to an individual's internal sense of self‐worth. It is also influenced by a variety of psychological, cultural and social factors. Furthermore, human dignity is unique to each person and is shaped by their relationships with others. Additionally, there is a sense that human dignity is a comprehensive concept that extends beyond the sum of its components.^11^, ^12^, ^13^

Maintaining patient dignity is a fundamental part of providing quality care.^14^ Patients recognize dignity as one of the most important aspects of care and make a direct connection between their sense of dignity and respect and the overall quality of their care.^15^ There is a strong relationship between preserving patient dignity and their satisfaction with care.^16^ Caregivers should be knowledgeable and skilled in how to preserve patient dignity in their work.^17^ Ensuring that patients are cared for while preserving their human dignity is a crucial element of healthcare delivery systems.^18^, ^19^, ^20^, ^21^

Preserving patient dignity includes upholding privacy, confidentiality, honest communication, involving the patient in their care, respecting their authority and control over the care process, respecting, viewing the patient as an individual and allowing them to make decisions.^21^ The importance of preserving human dignity and status has been highlighted in the 1984 General Assembly Declaration.^22^ The World Health Organization has also recognized human dignity as a key factor in promoting patient health, and identified the right to informed consent, access to health services, confidentiality of information and privacy as the most important rights in a 1994 Declaration.^22^

In recent years, considerable effort has been devoted to planning and policy‐making to improve the delivery of mental health care globally. However, the experience of all stakeholders has not been sufficiently considered. Enhancing the quality of care and sustaining the dignity of patients with mental health disorders requires examining how to improve care across a range of scenarios, from the mild to the most extreme. Therefore, this study aimed to explore the experience of patients, caregivers and family members/companions in preserving dignity during hospitalization in the mental health department.

## 2 MATERIALS AND METHODS

This study used a qualitative research approach. Participants were selected through purposeful sampling to meet the research objectives. The participants were patients, caregivers and family members of patients with mental health disorders hospitalized in a psychiatric ward of a University Medical Center over the past year. Patients were selected in consultation with psychiatrists. Inclusion criteria for patients were stable mental condition and ability to provide informed consent. Psychiatrists and nurses had at least 6 months of experience in the psychiatric ward. Family members were first‐degree relatives present during admission and regularly through the hospitalization.

Data were collected through face‐to‐face focus groups and face‐to‐face semistructured interviews based on an initial interview guide. Patients were asked about hospitalization experiences, experiences maintaining and harming dignity, and factors impacting dignity. Caregivers were asked about experiences in the psychiatric ward, hospitalizing psychiatric patients, situations preserving or damaging patient dignity and factors impacting dignity. Family members were asked about experiences during admission and hospitalization, situations preserving or damaging patient dignity and factors impacting dignity. Twenty‐six participants were interviewed, with one interviewed twice. Interviews lasted 24—63 min. Two focus groups of 83 and 74 min were conducted with the same family members. Conducting a focus group discussion is an effective approach to obtaining detailed insights into the perspectives and viewpoints of a community regarding a particular subject matter.^23^ Preserving the dignity of study participants, including patients and their families, was a primary focus throughout the data‐gathering process in our study. The participants were selected with the assistance of their respective psychiatrists, who first explained the study's purpose and nature to them. Following this, patients and their guardians provided written informed consent. Throughout the data gathering and analysis phases, the participants were referred to as study colleagues and addressed by their appropriate title and last name as a sign of respect. We communicated to them that their contribution was highly valuable and that their involvement was greatly appreciated, emphasizing the importance of their dignity and worth throughout the study.

Questions were refined based on emerging themes and categories. Probing questions clarified responses. Interviews were transcribed verbatim. Thematic analysis identified related features and subthemes until saturation, when new data matched existing data and no new data was added. The data were analysed through a combination of paper and pen methods, aided by Microsoft Excel 2016. The study participants were actively engaged in both data collection and analysis. They were given access to the transcribed interviews and focus group discussions and asked to provide input on the codes, categories and overarching themes. Their feedback was incorporated in the selection of relevant quotes, and they also provided commentary on the main findings of the study. Additionally, their insights were helpful in clarifying any misunderstandings that may have arisen during the data gathering and analysis process.

Ethical approval was obtained from the Iran University of Medical Sciences Biomedical Research Ethics Committee (IR.IUMS.REC.1395.121). Informed consent was obtained from participants and guardians. Confidentiality and anonymity were ensured using aliases. Findings were provided to relevant centres with proper referencing.

## 3 RESULTS

Upon completing data collection, 27 interviews and 2 focus group discussions took place. The participants consisted of 11 psychiatrists, 3 psychologists, 4 psychiatric nurses, 8 patients and 1 family member of the patients. Seven family members or companions participated in the focus group discussions.

The primary theme of this study was the violation of patient dignity due to negative guardianship. The subthemes identified were negative guardianship, custody and deprivation of rights. The results revealed that psychiatric illnesses impact a patient's dignity, regardless of the severity of the illness. Therapists may unintentionally violate the dignity of patients with mental health disorders because of their sense of guardianship, which threatens the patient's dignity. Table 1 presents the main theme, subthemes and primary codes of the study.

**Table 1:** Main theme, subthemes and main codes of the study.

Main theme	Subthemes	Codes
Violation of patient dignity through negative guardianship	Negative guardianship	Not being able to make decisions for oneself
		Waiting for a decision by the therapist on behalf of the patient or family
		There was no participatory approach Negative family reaction to being consulted by a therapist
		Consult with the family about the patient's discretion
	Dehumanization, namelessness and worthlessness	Not looking like a human being
		Not being respected
		A sense of worthlessness
		Feeling less than a human being
		Removal respectful titles
		Removal of the title to create empathy and reduce formality
	Violation of patients' rights	Talk about the illness with family
		Not being aware of your rights
		Not knowing the diagnosis
		Lack of insight into the patient at the time of hospitalization
		Not keeping patient information confidential
		Physical abuse
		Not getting patient consent
		Unprincipled hospitalization of the patient
	Deprivation patients of their authority	Hospitalization against the patient's wishes
		Ignoring patients' decision
		Ward restrictions
		Deprivation of the right to choose a therapist
		Ignoring the patient's wishes
		Lack of access to the main therapist

### 3.1 Meaning of dignity

At the start of the interview, the participants were asked to define the concept of dignity, and their responses were as follows:

*Patient, a 33‐year‐old single male*, believes that dignity is the genuine respect that every person is entitled to.

*Patient, a 28‐year‐old single female*, views dignity as being treated with respect and as a healthy human being, equal to others.

*Patient, a 46‐year‐old divorced male*, draws from his personal experience to define dignity as being trusted and receiving the respect one deserves.

*The psychiatrist, a 61‐year‐old female*, considers dignity to be the foundation of care, where both the patient and healthcare provider should be respected to achieve a healthy life.

*The psychologist, a 40‐year‐old female*, sees dignity as a more comprehensive and detailed form of respect.

*Lastly, the psychologist nurse, a 31‐year‐old female*, defines dignity as the core principle of care where the patient is viewed as a deserving and respected human being who should receive quality care.

They saw dignity as a wide and comprehensive concept related to respect and being treated respectfully.

### 3.2 Negative guardianship

The first theme was negative guardianship. Participants believed that a reason for overlooking patients' dignity is the sense of negative guardianship that some therapists have toward patients. Therapists thought that patients with psychiatric disorders were incapable of making their own decisions or might be exploited for various reasons.

*Psychiatrist*: ‘There have been cases where the family or relatives wanted to take advantage of the situation because of the patient's position, here the psychiatrist has a responsibility to defend the patient's rights’.

*Psychiatrist*: ‘One of the reasons our colleagues are so sensitive to these patients' rights is that they may make decisions that are detrimental to them or do something that affects their lives’.

*Psychiatric nurse*: ‘Many patients … do not have an insight into their disease and we are responsible for them’.

Healthcare providers often believe that patients cannot make decisions independently. Some therapists thought that asking the patient for their opinion would usually result in a negative response from the patient or their family, who expected the therapist to decide for them. In this respect, therapists believed they were better positioned to act in the best interest of patients who cannot act in their own best interests.

*Psychiatrist*: ‘If we ask them, the situation will get worse. They say we came here for your guidance, But I see you know nothing’.

*Patient*: ‘I expect my therapist to tell me what to do, if he does not tell me, what am I going to do?’

*Companion*: ‘We mostly expect him to be treated. We do not know anything about the treatment and they did not provide enough information so we go to someone who knows better than us’.

This decision‐making process evolved from a sense of guardianship that regards patients as incapacitated individuals who are incapable of having any rights and are deprived of self‐determination and decision‐making.

*Inpatient*: ‘I consult with my doctor or psychologist about most issues in my life because I think I made many mistakes in my life that would not have happened if I had consulted’.

Therapists, patients and family members or companions expected the psychiatrist to have the authority to make decisions about all aspects of the patient's life, making the participatory approach invisible.

*Psychiatrist*: ‘We are asked whether he should be hospitalized or not, or they even ask if he (should) get married or not. We usually answer them according to the patient's condition, it is less common to ask their opinion’.

*Companion*: ‘In the case of patients like ours, the doctor knows better what is better for him. We also trust him and it has been good so far’.

*Psychiatric nurse*: ‘It is rare for a patient to be asked what he wants. Usually, his family makes decisions in consultation with a doctor’.

Decision‐making on behalf of patients at a highly personal level was also not uncommon. Depriving a person of essential life decisions, such as choosing to marry or having relationships with others, was one aspect that did not occur during hospitalization. However, questions about these issues from the patient and their families at the time of hospitalization were not usually well answered. Some psychiatrists spent more time answering the patient's questions in this regard.

*Psychiatrist*: ‘They ask us if he should get married. They also believe that if he gets married, it will be fine. I tell them that the other party must be aware of this person's condition’.

*Companion*: ‘We ask the doctor whether he should get married or not, but usually, their answer is not straightforward’.

Many crucial life decisions may be taken away from patients and given to decision‐makers like psychiatrists. In this manner, the patient is deprived of their rights, infantilized and unable to make critical life choices.

Decisions about who should be informed of the patient's diagnosis were also less frequently made in consultation with patients.

*Patient*: ‘They did not ask my opinion whether to tell my family or not, … I was not good enough to decide. My family asked and they were told that this was probably the diagnosis’.

*Psychiatrist*: ‘When the family asks and the patient cannot make a decision, I usually tell them’.

*Psychiatrist*: ‘There are restrictions, for example, we are careful to tell their spouse’.

*Assistant*: ‘Usually, the family insists on knowing the diagnosis, and we tell them the diagnosis; we typically do not ask the patient’.

### 3.3 Dehumanization, namelessness and worthlessness

Negative guardianship may lead to some degree of infantilization, depriving patients of their authority and ability to self‐determine or collaborate in decision‐making. This can result in feelings of dehumanization, anonymity and worthlessness, as experienced and observed by patients, practitioners and companions.

*Patient*: ‘They referred to themselves as Mr. Doctor or Mrs. Nurse, but they addressed us by our first or last name without any titles like Mr. or Mrs.’.

*Companion*: ‘Not all, but some staff members don't show much respect to patients’.

Participants believed that these behaviours made them feel less than human. They mentioned not being addressed by their usual titles.

*Patient*: ‘The person in the hospital might have had a job before; I was employed. In the hospital, they called me by my last name only and talked down to me like I didn't understand anything, as if I were a child’.

This lack of respect, where patients are not given their due titles and are belittled, makes them feel disrespected, dehumanized and infantilized.

*Companion*: ‘Generally, the treatment was respectful, but one could sense that they see the patient as lacking a complete personality like other people’.

Some psychiatrists and other healthcare teams believed that calling patients by their first name created empathy and intimacy. Others strongly opposed this practice and aimed to create empathy while maintaining the patient's respect.

*Psychiatrist*: ‘I believe it's disrespectful to call patients loudly from the waiting room’.

*Psychiatrist*: ‘I address patients by their titles and ask my assistants to do the same. I prefer being called by my title as well. I think it's the right way, but I see patients being called without a title. Maybe they want to get closer and create empathy’.

*Psychiatrist*: ‘If we call patients without titles, it's to lessen the formality and distance between us’.

*Nurse*: ‘In our department, calling patients without titles happens frequently, but it's meant to create intimacy’.

*Psychologist*: ‘I take it very seriously, but I've seen it happen. I think it's to strengthen empathy, but I don't find it appropriate’.

All three interviewed groups reported cases where patients' dignity was not observed during admission. The results revealed that patient dignity is a sensitive issue that many therapists pay special attention to, but hospital conditions and patients' lack of rights awareness make it challenging to respectfully. Although practitioners claimed to take this matter seriously, this was less evident from patients' and companions' perspectives:

*Psychologist*: ‘I always invite patients into the room or stand up when they arrive. I escort them out when they leave, giving them a sense of respect and dignity’.

*Psychologist*: ‘I explain patients' rights to all of them; this is one of the most important ways to respect their dignity’.

*Nurse*: ‘All our doctors show the utmost respect for patients and set a great example. I've never seen a case of patient disrespect.

*Companion*: ‘The overall treatment is good, and we're satisfied, but our patient's condition is special. They need more respect’.

*Patient*: ‘I've been hospitalized several times. The level of respect varies depending on the place, doctor and ward. I've felt both respected and unworthy as a person’.

There is a notable disparity in perceptions between practitioners and patients or companions concerning respect and honour, particularly in the theme of rights deprivation.

### 3.4 Violation of patients' rights: Rights to diagnosis knowledge, care choice, confidentiality and freedom from harm

Disrespecting the dignity of psychiatric patients often manifests as rights deprivation, particularly concerning their right to know their diagnosis. Psychiatrists usually prefer not to disclose the diagnosis to psychiatric patients for various reasons.

*Patient*: ‘For a long time, I didn't know my diagnosis. I only knew I had a mental illness and needed medication. My doctor eventually told me I had a mood disorder. I didn't follow up until they finally told me I was bipolar. Then I researched and learned what it meant’.

*Companion*: ‘We only knew that the patient had a psychiatric disorder; we didn't know the diagnosis’.

*Psychiatrist*: ‘Knowing the diagnosis alone doesn't help. They may search the internet, and it could confuse them’.

Patients' experiences also showed that they were not informed of their diagnosis even after a long hospitalization.

*Patient*: ‘I was hospitalized several times and took many medications, but I didn't know my disease's name’.

Psychiatrists cited several reasons for not disclosing the diagnosis, including the patient's lack of insight at admission, inability to understand the diagnosis and the patient not asking for it.

*Psychiatrist*: ‘I don't think knowing the diagnosis is very helpful, but if they ask, I'll tell them. There should be a system that provides the necessary education’.

Receiving other basic rights, such as choosing a therapist during hospitalization, was also reported. This was mostly related to the structure of patients' admissions during emergency visits.

*Patient*: ‘Dr … was my doctor, but when you come to the emergency room, you cannot choose your doctor. You are sent to another doctor; this makes me sad’.

Instances of physical abuse, lack of privacy and confidentiality were also reported:

*Psychiatrist*: ‘There were cases where health staff hit patients; we saw the video, and those staff members were punished’.

*Psychiatrist*: ‘Like how maternal cases shouldn't be examined in front of students, we're doing the same with psychiatric patients. We don't ask for the patient's consent while ten students are sitting next to us. Would you be comfortable if I asked you this question?’

One of the patients' rights that were often overlooked was the right to confidentiality. Patient information was frequently shared with family members without consulting the patient, especially during emergencies. This was more likely when therapists were questioned by the family.

*Psychiatrist*: ‘I usually tell the family because the patient doesn't have any insight, and someone has to decide’.

*Patient*: ‘I didn't want my wife to know my diagnosis. I don't know how she found out, but it wasn't good for me’.

### 3.5 Depriving patients of their authority

One crucial aspect of diminished dignity for psychiatric patients is the loss of their authority. This study's findings indicate that psychiatric patients often lose their decision‐making power on numerous matters in their lives. A key aspect is the inability to make decisions regarding their treatment process.

*Psychiatrist*: ‘Patients with mental health disorders sometimes lack the authority to make decisions. They may struggle with decision‐making, but we sometimes forget they can still make choices for themselves. For instance, a patient may say they don't want medication for their depression, and I might respond that if they don't want medication, they shouldn't come to me for help. Their manner of expression might not be respectful, but they're being honest. I don't offer psychotherapy, so I refer them elsewhere. If they don't follow my advice, I might get frustrated and refuse to see them anymore. I rarely see them asking about their preferred treatment options’.

Regarding hospitalization, patients mentioned that even when they had the authority to decide on their admission or were aware of their condition and did not want to be admitted, they were hospitalized against their will.

*Patient*: ‘I knew I wasn't very sick, but when I argued with my brother, nobody listened to me and they took me to the hospital. No matter what I said, they got angry and put me to bed. I wasn't hospitalized for long and was discharged soon’.

In such cases, doctors also acknowledged that they sometimes had to treat patients due to family pressure, even when the patient was capable of making their own decisions. This situation exemplifies the deprivation of patient authority. Most of these forced hospitalizations occurred during night shifts by assistants.

*Psychiatrist*: ‘The patient wasn't very sick, but the family insisted they were in a lot of pain. We admitted them, and they were discharged the following morning’.

Psychiatric patients also encountered challenges regarding admission, such as obtaining permission to leave the ward, not being allowed to have a cell phone on the ward, and needing approval for simple tasks. These restrictions exemplify the disenfranchisement they experience.

*Psychiatrist*: ‘For instance, we take away their mobile phones. However, this patient is no different from other patients’.

*Psychiatrist*: ‘Initially, there was significant resistance to allowing them to keep their mobile phones, but I advocated for their right to have them. Eventually, it became clear there was no issue’.

## 4 DISCUSSION

The current study's findings indicate that the dignity of psychiatric patients during hospitalization is not adequately addressed as outlined in the Charter of Patients' Rights. Our results reveal that healthcare providers exhibit a negative guardianship towards patients, leading to dehumanization, depersonalization, violation of patients' rights and stripping of patients of their authority. Consequently, patients in mental health facilities may suffer from a lack of dignity maintenance, negatively impacting the quality of care.

Our findings align with previous research. In a study by Chambers et al., 19 adults in a mental health unit in the United Kingdom were surveyed. Patients' experiences revealed that their dignity was impacted by factors including staff not listening to their concerns, lack of participation in treatment and care decisions, insufficient information about treatment plans and medications, limited access to therapists and an unsupportive physical environment for physical activities.^6^ We also found that lack of participation in the decision‐making and treatment violates the patient's dignity. This result was also supported by Scorpen et al. who conducted a study examining the experiences of patients, relatives and caregivers regarding patients' dignity in mental health departments. Their primary resulted theme was ‘the importance of small things for experiencing dignity’, with subthemes encompassing awareness of minor issues, a consciousness of spoken words and satisfaction and recognition of interpersonal relationships. Employees' behaviour directly impacted patients' and their companions' perception of dignity. When patients and families are treated with values such as equality and respect, imbalances in relationships can cause resentment.^24^, ^25^ Our results also showed that patients should be treated respectfully. They need to be seen as human and they compare themselves with patients with physical illnesses.

In 2012, Lindwall et al. published findings on preserving patients' dignity in psychiatric wards. This study aimed to describe nurses' experiences in handling situations related to patients' dignity in psychiatric wards. Findings demonstrated that when caregivers work according to their moral duty, patient dignity is preserved.^26^ Gastfson's study identified seven primary themes: patients not being taken seriously, patients being ignored, disclosure of patients' secrets, violence against patients, victimization of patients, abuse of patients' trust and predefining patients.^27^ Their results were in line with our study. We also found that disclosure of patients' diagnoses to their families and ignoring them made them feel undignified.

Maintaining a patient's dignity fosters a sense of comfort, confidence and worth, which can aid in treatment and care decisions. Conversely, when a patient's dignity is neglected, feelings of uncertainty, humiliation and shame can adversely affect treatment and care outcomes.^28^ The foundation of care is respect for individual dignity, which is increasingly important every day.^22^ Dignity is a fundamental concept in health systems and the focus of medical and nursing care.^29^ In medical environments and hospitals, dignity encompasses independence, honesty, justice, respect for human rights, awareness and active defence of the patient.^30^

Preserving patient dignity in various hospital wards is crucial, and respecting rights related to individual dignity is a principle of work and professional ethics. Some studies have shown that hospital patients are susceptible to losing their human dignity.^31^ Regrettably, evidence suggests that most healthcare workers view the world solely from their professional perspective, limiting their thinking, judgement and ultimately their performance. Expanding their understanding of the phenomenon in various ways is essential to achieving optimal performance.^32^ The history of the healthcare system introduction demonstrates a shift from a biomedical approach focused on disease, signs and symptoms to a holistic approach emphasizing human values and experiences.^26^ Treatment for patients with mental disorders may be performed without their consent, at the therapist's discretion and based on the principle of utility. The ability to participate in treatment decisions, have authority, provide informed consent and maintain confidentiality is sometimes overlooked by patients admitted to mental health wards.^33^ This negligence can result in a sense of lost authority and helplessness in the patient, negatively affecting their recovery.^33^, ^34^, ^35^ Furthermore, research has shown that a loss of patient dignity may even diminish their desire for recovery and survival.^36^

Enhancing human dignity is thus a critical consideration. Improving human dignity in medical care users can increase patient self‐confidence while disrespecting dignity can lead to severe forms of physical and mental deterioration.^31^, ^37^, ^38^ When a person with a mental disorder is admitted, stress can manifest as physical behaviour that may harm oneself or others.

### 4.1 Limitations

While our findings offer valuable insights into dignity within mental health settings and hospitalization, further research on the dignity of patients with mental health disorders in various contexts, such as family and workplace settings, would be beneficial.

## 5 CONCLUSION

Our study underscores the importance of conducting further research on the concept of dignity in varied settings to enhance our comprehension of it. We also recommend the development of quantitative measures to evaluate the preservation of dignity across multiple perspectives. Based on our findings, we suggest that psychiatrists, psychologists and nurses, who are at the forefront of providing mental health care, require additional education and improved attitudes toward dignity in mental health settings. They must view the care and treatment system from the patient's perspective and gain a deeper understanding of the significance of dignity and respect in enhancing patients' quality of life and well‐being. One of the primary reasons for compromised dignity in mental health settings is the negative attitudes of healthcare providers toward individuals with mental health disorders. They must adopt a more positive outlook toward the abilities and lives of these patients. Our study reveals that despite the efforts of psychiatrists and therapists to empathize and safeguard patients' rights, the nature of mental health disorders causes some hospital staff to perceive these individuals as incompetent to make critical life decisions. This perspective leads therapists to make decisions on behalf of their patients, resulting in a disregard for their rights and a loss of decision‐making authority.

## AUTHOR CONTRIBUTIONS

H.R., A.S., C.B., and Y.R. wrote the manuscript draft. H.R. and F.A. designed the study. F.A., A.A. and M.A. did the data gathering. H.R., A.S., and C.B. conducted the analyses. All authors reviewed the final manuscript.

## CONFLICT OF INTEREST STATEMENT

The authors declare no conflict of interest.

## Data Availability

Data are available on request due to privacy/ethical restrictions.
